# Sortin2 enhances endocytic trafficking towards the vacuole in *Saccharomyces cerevisiae*

**DOI:** 10.1186/s40659-015-0032-9

**Published:** 2015-07-25

**Authors:** Beatriz Vásquez-Soto, Nicolás Manríquez, Mirna Cruz-Amaya, Jan Zouhar, Natasha V Raikhel, Lorena Norambuena

**Affiliations:** Plant Molecular Biology Centre, Department of Biology, Faculty of Sciences, University of Chile, Las Palmeras 3425 Ñuñoa, Santiago, Chile; Centro de Biotecnología y Genómica de Plantas, Universidad Politécnica de Madrid, Madrid, Spain; Center for Plant Cell Biology, Department of Botany and Plant Sciences, University of California, Riverside, CA 92521 USA

**Keywords:** Endocytosis, Sortin2, Trafficking modulator, *Saccharomyces cerevisiae*

## Abstract

**Background:**

A highly regulated trafficking of cargo vesicles in eukaryotes performs protein delivery to a variety of cellular compartments of endomembrane system. The two main routes, the secretory and the endocytic pathways have pivotal functions in uni- and multi-cellular organisms. Protein delivery and targeting includes cargo recognition, vesicle formation and fusion. Developing new tools to modulate protein trafficking allows better understanding the endomembrane system mechanisms and their regulation. The compound Sortin2 has been described as a protein trafficking modulator affecting targeting of the vacuolar protein carboxypeptidase Y (CPY), triggering its secretion in *Saccharomyces cerevisiae*.

**Results:**

A reverse chemical-genetics approach was used to identify key proteins for Sortin2 bioactivity. A genome-wide Sortin2 resistance screen revealed six yeast deletion mutants that do not secrete CPY when grown at Sortin2 condition where the parental strain does: *met18*, *sla1*, *clc1*, *dfg10*, *dpl1* and *yjl175w*. Integrating mutant phenotype and gene ontology annotation of the corresponding genes and their interactome pointed towards a high representation of genes involved in the endocytic process. In wild type yeast endocytosis towards the vacuole was faster in presence of Sortin2, which further validates the data of the genome-wide screen. This effect of Sortin2 depends on structural features of the molecule, suggesting compound specificity. Sortin2 did not affect endocytic trafficking in Sortin2-resistant mutants, strongly suggesting that the Sortin2 effects on the secretory and endocytic pathways are linked.

**Conclusions:**

Overall, the results reveal that Sortin2 enhances the endocytic transport pathway in *Saccharomyces cerevisiae*. This cellular effect is most likely at the level where secretory and endocytic pathways are merged. Them Sortin2 specificity over the endomembrane system places it as a powerful biological modulator for cell biology.

**Electronic supplementary material:**

The online version of this article (doi:10.1186/s40659-015-0032-9) contains supplementary material, which is available to authorized users.

## Background

Eukaryotes have developed an intricate system of protein delivery to various cellular compartments, based on highly regulated trafficking of cargo vesicles. Cargo recognition, vesicle formation and fusion represent the core of endomembrane system processes and form two main routes, the secretory and the endocytic pathways. These two pathways have pivotal functions in uni- and multi-cellular organisms. New discoveries have highlighted the role of the endomembrane system in diverse cellular processes such as cell polarity, signaling, development and response to environmental challenges [1, 2]. Therefore, new and innovative tools to manipulate endomembrane trafficking are of great interest.

Chemical genomics is a powerful tool to discover such new biomodulators. It employs large diverse collections of compounds to identify small bioactive molecules in order to manipulate biological pathways in a similar fashion to classical genetics [3, 4]. The bioactive compound may be used as an instrumental tool to manipulate biological processes even when the cognate target(s) is(are) still unknown. The identification of the members of drug-sensitive pathways may aid in the identification and characterization of novel intracellular networks. Successful approaches for such duty have included biochemical and genetic analysis [5, 6].

Genetic approaches are usually carried out by genome-wide screens that involve searching through collections of mutants for those with altered sensitivities to the drug, i.e., hypersensitivity or resistance. The most used model to carry out such screenings has been *Saccharomyces cerevisiae* deletion mutant collections, which have led to the identification of several novel components of cellular networks [7–9].

Drugs that impair vacuolar trafficking in *S. cerevisiae* have been identified and called sorting inhibitors (Sortins) [10]. These compounds mimic the phenotype of *vacuolar protein sorting* (*vps*) mutants [11–13] suggesting that they modify components of the endomembrane trafficking pathway. A genome-wide hypersensitivity screen showed that Sortin2 affects primarily components within the endomembrane system in yeast [14]. This analysis identified 217 Sortin2 hypersensitive Saccharomyces deletion mutants in where the mutated genes are enrichment on genes products involved on protein trafficking and localized particularly in endosomes [14]. Therefore the Sortin2 mode of action and the proteins involved on it are interesting subjects of study especially for further uses of the drug.

In order to identify proteins that are critical for the mode of action of Sortin2, we used a reverse chemical-genetics approach; we performed a Sortin2-resistant genome-wide screening using a *S. cerevisiae* haploid deletion library. We found six ORFs whose deletion caused Sortin2 resistance. Mutant phenotypes, GO annotation and the interactome of the corresponding genes indicated endocytosis as the principal GO process. Consistently, Sortin2 treatment enhanced trafficking of the endocytic tracer FM4-64 toward the vacuole. Analysis of structure–bioactivity relationships suggested that Sortin2 effects on endocytosis toward the vacuole depend on Sortin2 structural features. Genetic and chemical analog analysis strongly suggests that the effect of Sortin2 on the secretory pathway is linked to its effect on endocytosis.

## Results

### Sortin2 resistance genome-wide screen

**Figure 1:**
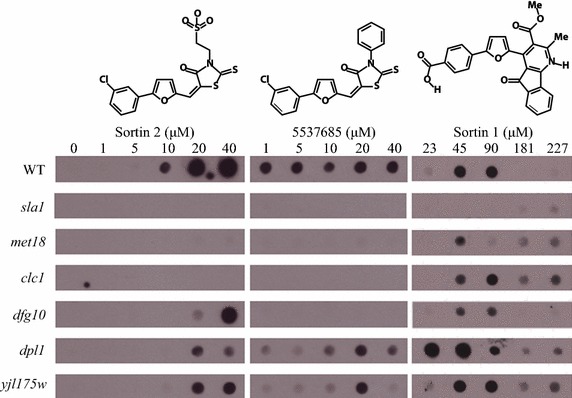
Sortin2 *S. cerevisiae* resistance is specific to Sortin2-related structures. Mutant and parental (WT) strains were grown in YPD medium supplemented with the indicated concentrations of Sortin2 (μM), compound 5537685 and Sortin1. The control condition (0 μM Sortin2) contained 1% DMSO, which is present in all conditions since it is the solvent for the three compounds. The presence of CPY was analyzed on the growth medium by dot-blot using a CPY monoclonal antibody. The experiment was performed three times.

In order to find important molecular component(s) for Sortin2 bioactivity in yeast, a screen for mutants resistant to Sortin2 was performed using a loss-of-function mutant collection of 4,800 haploid deletion *S. cerevisiae* strains. It was anticipated that this resistance screening would identify proteins required for Sortin2 bioactivity that were not codified by an essential gene. The parental strain secretes CPY when grown with 10 μM Sortin2 [14]. The primary screen was performed with Sortin2 47 μM that is almost five times higher of the minimal concentration that trigger CPY secretion in the wild type strain. Therefore the screening could identify the strong resistance to Sortin2. Out of the entire collection, 36 putative resistant strains were identified (Additional file 1: Table S1). These strains were retested with different concentrations of Sortin2 to confirm the resistant phenotype. The threshold to consider a strain resistant to Sortin2 was 10 μM because that was the concentration that trigger secretion of CPY in the wild type strain using peroxidase detection. Mutant strains that did not secrete CPY with 10 μM Sortin2 were considered as Sortin2-resistant mutants. Out of the 36 primary screen hits, 6 mutants were confirmed as resistant to Sortin2 by this dosage dependence analysis (Figure 1; Additional file 1: Table S1). The results showed that *sla1*, *met18* and *clc1* were resistant to up to 40 µM Sortin2. Furthermore *dfg10* and *dpl1* were resistant to Sortin2 up to 20 µM as well as the dubious mutant *yjl175w*. Sortin2 did not inhibit growth performance of the Sortin2-resistant mutants, which was evaluated by growing them in 47 µM of Sortin2 for 10 h (Additional file 2: Figure S1). Sortin2 does not inhibit the growth neither the viability of five of the Sortin2-resistant mutants when they grown for 72 h with 20 µM of the chemical (Additional file 3: Figure S2). Therefore, the observed resistance to Sortin2 of *met18*, *sla1*, *clc1*, *dfg10* and *dpl1* was not due to cell growth impairment but rather to changes in trafficking machinery that resulted in reduced secretion of CPY. In the case of *yjl175w*, which deletion affects a dubious gene, this condition of treatment inhibits its growth (Additional file 3: Figure S2). Therefore the lack of CPY secretion resistance to Sortin2 could be due to a lower amount of cells and CPY detection limits. Interestingly Sortin2 increases the viability of *met18* and *dpl1* suggesting than this compound improve cell performance (Additional file 3: Figure S2).

CPY is a vacuolar soluble protein that is secreted to the extracellular medium under certain chemical or genetic conditions [10, 15, 16]. Therefore, the Sortin2 resistance observed in *sla1*, *met18* and *clc1* could be due to general defects in the secretory pathway in the mutant background. To test this hypothesis, Sortin1, which also induces secretion of CPY [10], was evaluated for its effect on CPY trafficking in identified resistant mutants. Sortin1 was able to trigger CPY secretion in the *sla1*, *met18* and *clc1* strains (Figure 1), suggesting that the mutants have a functional secretion pathway. Although Sortin1 triggers CPY secretion in *sla1* in much less extension than the parental strain the sensitivity of *sla1* to three different CPY-secretion triggering compounds confirms its ability to secrete CPY (Additional file 4: Figure S3). Importantly, Sortin1 treatment induced CPY-mistargeting in Sortin2-resistant mutants, indicating that the corresponding deletion confers resistance to certain structural determinants of Sortin2. Furthermore, *sla1*, *met18*, *clc1* and *dfg10* were also resistant to the Sortin2 structural analog 5537685, revealing that Sortin2 resistance is specific for Sortin2-related structures in these strains (Figure 1). On the contrary, *dpl1* and yjl175w were sensitive to 5537685 although their secretion was much weaker than the parental line. Therefore the Sortin2 resistance of these two mutants could be due to structural differences between Sortin2 and 5537685 (Figure 1).

We further analyzed the gene ontology term representation for genes whose deletion provokes resistance to Sortin2. According to the GO annotation available in the *S. cerevisiae* database (SDG, http://www.yeastgenome.org/), four of those genes encode proteins that are related to endomembrane trafficking: CLC1, SLA1, DFG10 and DPL1 (Table 1). This finding is consistent with the specific mode of action of Sortin2 on the yeast endomembrane system [14]. The CLC1 and SLA1 genes encode a clathrin light chain and an actin binding protein, respectively, and are directly involved in endocytosis [17, 24]. In addition, *sla1*, *met18*, *dfg10* and *yjl175w* mutants show endocytosis defects, indicating that the encoding proteins are important for this cellular process [39]. The YJL175W gene corresponds to a dubious ORF but its deletion also affects endocytosis [39]. Therefore among the genes that encode proteins required for Sortin2 bioactivity, five out of six genes participate in the endocytosis process.

**Table 1:** Gene ontology (GO) of genes whose deletion provokes resistance to Sortin2 in *S. cerevisiae*

Gene/ORF	GO function	GO process	GO component	References
SLA1 YBL007C	Protein binding, bridging^1^ Ubiquitin binding^2^ Actin binding^3^ Cytoskeletal protein binding^4^ Identical protein binding^4^	Actin cortical patch assembly^5^ Endocytosis^6^ Fungal-type cell wall organization^5^	Actin cortical patch^7^ Cell cortex^1^ Nucleus^8^ Mating projection tip^9^ Cytoskeleton^3^ Endosome membrane^3^ Plasma membrane^3^	^1^Warren et al. [17] ^2^Stamenova et al. [18] ^3^UniProt-GOA [19] ^4^DDB et al. [20] ^5^Pruyne and Bretscher [21] ^6^Howard et al. [22] ^7^Ayscough et al. [23] ^8^Gardiner et al. [24] ^9^Narayanaswamy et al. [25]
CLC1 YGR167W	Structural molecule activity^1^ Calmodulin binding^2^ Structural molecule activity^3^	Endocytosis^4^ Vesicle-mediated Transport^1,3^ Intracellular protein transport^3^	Clathrin vesicle coat^1^ Clathrin coat of coated pit^3^ Clathrin coat of trans-Golgi network vesicle^3^	^1^Pishvaee et al. [26] ^2^UniProt-GOA [19] ^3^DDB et al. [20] ^4^Newpher and Lemmon [27]
MET18 YIL128W	Binding^5^	Methionine metabolic process^1^ Nucleotide-excision repair^2^ Transcription from RNA polymerase II promoter^3^ DNA repair^6^ Response to DNA damage stimulus^6^ Transcription, DNA-dependent^6^	Cytoplasm^4^ Nucleus^6^	^1^Masselot and De Robichon-Szulmajster [28] ^2^Kou et al. [29] ^3^Lauder et al. [30] ^4^Huh et al. [31] ^5^DDB et al. [20] ^6^UniProt-GOA [19]
DFG10 YIL049W	3-Oxo-5-alpha-steroid 4-dehydrogenase activity^1^ Oxidoreductase activity, acting on the CH–CH group of donors^5^	Dolichol biosynthetic process^1^ Pseudohyphal growth^2^ Lipid metabolic process^5^	Integral to membrane^3,4,5^ Endoplasmic reticulum membrane^4^	^1^Cantagrel et al. [32] ^2^Mosch and Fink [33] ^3^De Hertogh et al. [34] ^4^UniProt-GOA [19] ^5^DDB et al. [20]
DPL1 YDR294C	Sphinganine-1-phosphate aldolase activity^1^ Carboxy-lyase activity^6^ Pyridoxal phosphate binding^6^	Calcium-mediated signaling^2^ Cellular response to starvation^3^ Sphingolipid metabolic process^1^ Carboxylic acid metabolic process^6^	Endoplasmic reticulum^4,5^	^1^Saba et al. [35] ^2^Birchwood et al. [36] ^3^Gottlieb et al. [37] ^4^Mukhopadhyay et al. [38] ^5^Huh et al. [31] ^6^DDB et al. [20]
YJL175W YJL175W	Unknown	Unknown	Integral to membrane^1^	^1^De Hertogh et al. [34]

Biological process, molecular function and cellular component GO are shown for each ORF.

### Sortin2 enhances endocytic trafficking toward the vacuole in *S. cerevisiae*

**Figure 2:**
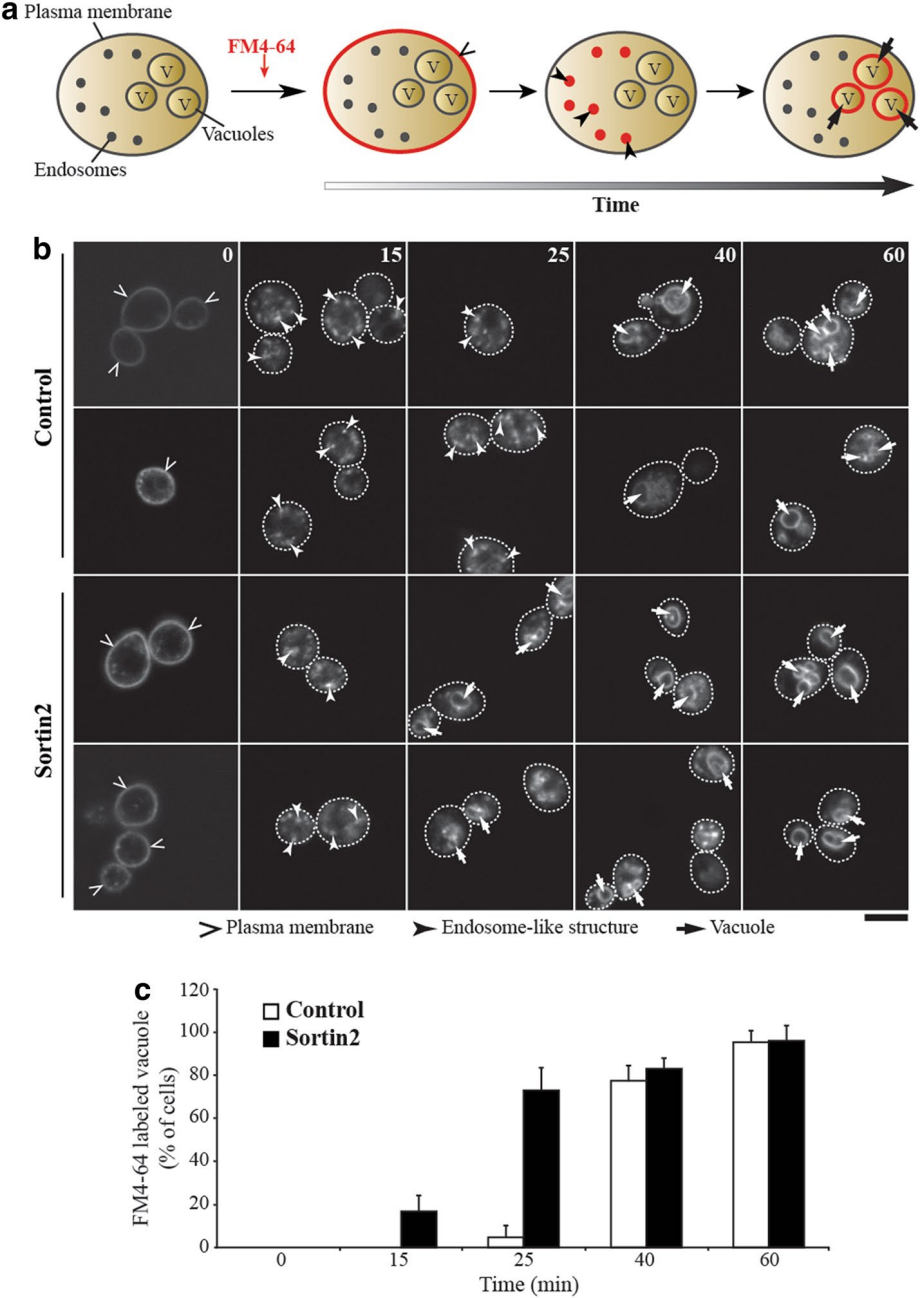
Sortin2 enhances internalization of FM4-64. **a** Diagram of FM4-64 endocytosis labeling. Cells were incubated with 24 μM FM4-64 for 30 min at 4°C for plasma membrane FM4-64 binding. Then turned to 28°C (time 0) to be imaged subsequently at different times of incubation by confocal microscopy. Progressively FM4-64 is progressively trafficking to intracellular compartments by endocytosis. **b** *S. cerevisiae* parental line was grown on YPD 1% DMSO (control) and YPD supplemented with 20 μM Sortin2. Two representative images of approximately 25 cells are shown in each condition. The experiment was performed three times. *Scale bar* represents 5 μm. **c** Cells with FM4-64 labeled vacuoles were scored in each condition on **b**. The percentage of cells with FM4-64 labeled vacuoles is informed with standard deviation.

Due to the functional characteristics of gene products affected in Sortin2-resistant mutants, the effect of Sortin2 in the endocytic route was analyzed. Internalization of the endocytic tracer FM4-64 [40] was analyzed by confocal microscopy (Figure 2a). In wild type *S. cerevisiae* (control), the FM4-64 dye reached the vacuole in approximately 40 min (Figure 2b, c). However, in cells treated with Sortin2, FM4-64 reached the vacuole in 25 min (Figure 2b, c), while in wild type cells the FM4-64 was still localized to endosome structures (Figure 2b). This result indicates that Sortin2 likely enhances endocytic trafficking towards the vacuole in *S. cerevisiae*.

**Figure 3:**
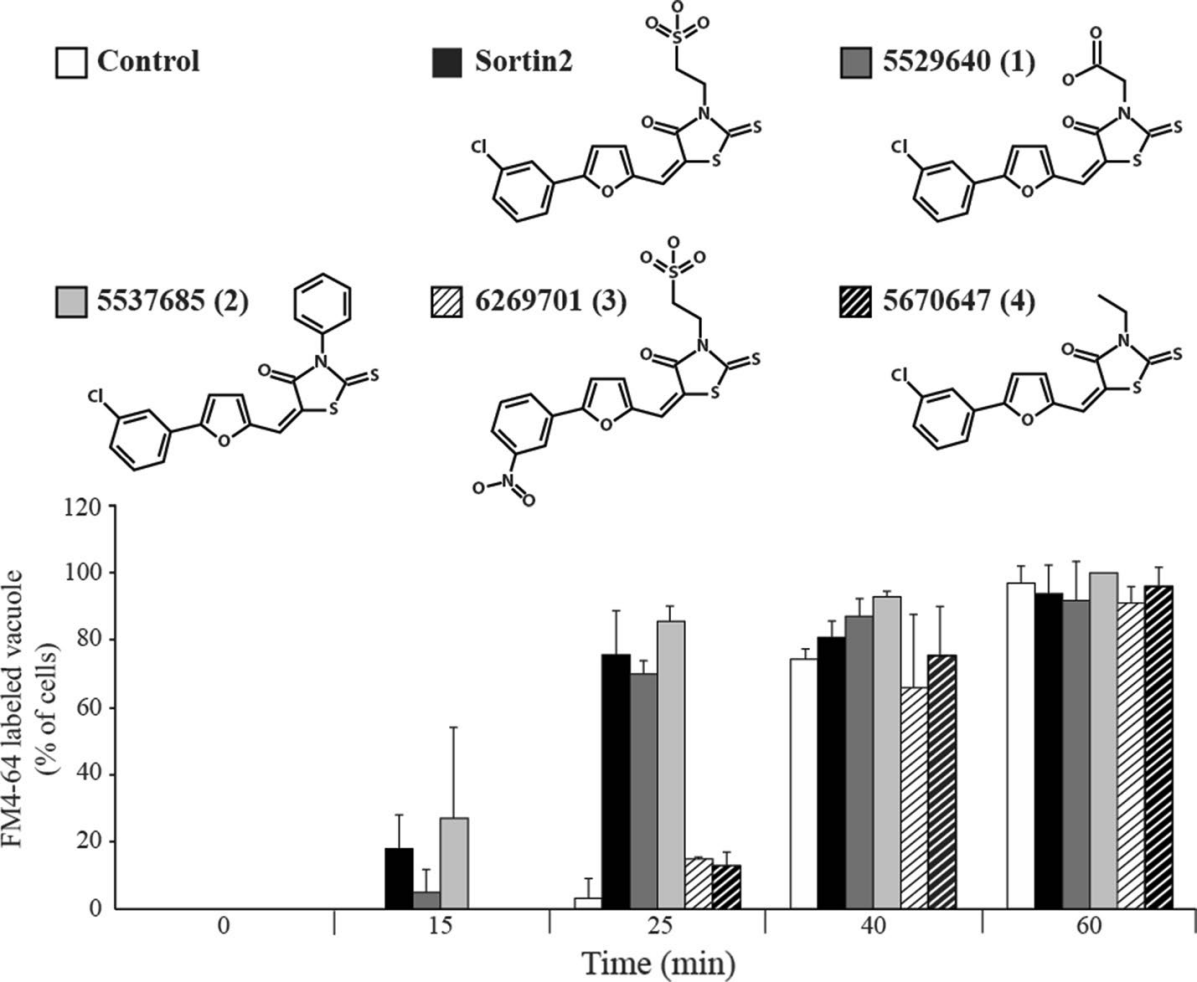
Enhancing of endocytic trafficking depends on Sortin2 structural features. *S. cerevisiae* parental line was grown on YPD 1% DMSO (control) and YPD supplemented with 20 μM of different Sortin2-structural analogs. Afterwards cells were incubated with 24 μM FM4-64 for 30 min at 4°C. Then turned to 28°C to be imaged subsequently by confocal microscopy at different incubation times. Cells with FM4-64 labeled vacuoles were scored. The number of cells with labeled vacuoles relative to the total scored population (N = 30 cells) in different times of FM4-64 incubation is informed with standard deviation.

To determine the specificity of the Sortin2 impact on endocytosis, the effect of compounds structurally related to Sortin2 was analyzed. The results showed that chemicals 5529640 and 5537685 speed up the FM4-64 endocytic pathway similarly to Sortin2 (Figure 3, Additional file 5: Figure S4). Therefore changing the sulfonate group in Sortin2 did not have an impact in its effect on endocytosis. Importantly, both 5670647 and 6269701 compounds had no effect on endocytosis timing, indicating that functional groups on both ends of Sortin2 molecule are important for Sortin2 bioactivity (Figure 3, Additional file 5: Figure S4). This result is consistent with the lack of activity of 5670647 and very low potency of 6269701 regarding CPY secretion [14].

### Sortin2 does not alter endocytic trafficking in Sortin2 resistant mutants

**Figure 4:**
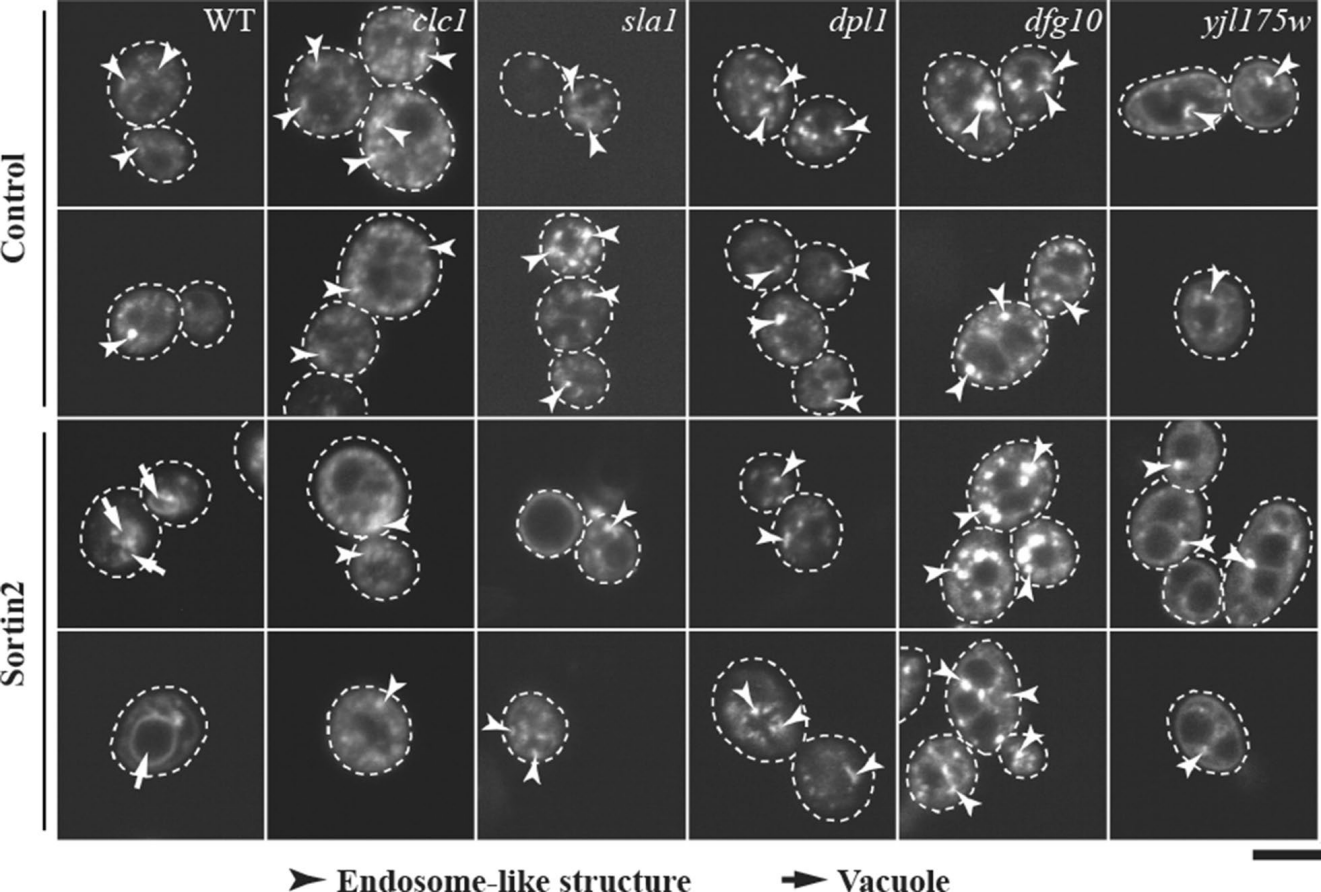
Sortin2 does not alter endocytic trafficking in Sortin2 resistant mutants. *S. cerevisiae* parental line (WT) and Sortin2 resistant mutants were grown on YPD supplemented with 1% DMSO (control) or 20 μM Sortin2. Cells were incubated with 24 μM FM4-64 for 30 min at 4°C. Then turned to 28°C to be imaged after 25 min by confocal microscopy. Images of 25 min incubation are shown. Two images are representative of 20 cells. The experiment was done more than three times. *Scale bar* represents 5 μm.

It has been demonstrated that Sortin2 not only impairs trafficking of CPY to the vacuole [10, 14] but also modifies the kinetics of endocytic trafficking in *Saccharomyces cerevisiae*. In order to test if the two phenotypes are linked, we analyzed whether Sortin2-resistant mutants were also resistant to the Sortin2 effect in endocytic trafficking toward the vacuole. Due to the size of the *met18* mutant cells and subsequent difficulties in assessing their subcellular phenotypes, the *met18* strain was not tested. Other Sortin2 resistant mutants were treated with Sortin2 at a concentration that made FM4-64 trafficking to the vacuole faster in the parental line (Figure 4). At 25 min FM4-64 labeled solely endosomes in all mutants treated with Sortin2, as in control conditions (Figure 4), indicating that mutations that confer Sortin2 resistance also suppressed the enhanced endocytosis phenotype observed for the wild-type strain. FM4-64 did not reach the vacuole at 25 min in any of the Sortin2-treated mutants. However, after 40 min of internalization FM4-64 was localized in the vacuole membrane, indicating that the lack of Sortin2 effect was not due to mutant impairment of endocytosis (Additional file 6: Figure S5).

### Interactome of proteins required for Sortin2 bioactivity

In order to determine whether identified gene products required for Sortin2 bioactivity are directly or indirectly involved in endocytic pathways, their interactome network was determined using OSPREY software [41]. We used the six gene products as query to build the interactome of physical and genetic interactions. The resulting network contained 273 nodes with edges representing physical or genetic interactions (data not shown). These nodes were classified according to their location and cell function using CellLoc and FunCat tools (http://www.mips.gsf.de), respectively. The Sortin2 resistance interactome analysis showed genes involved in different cellular process. The largest enrichment processes were “Cell fate” and “Cell type differentiation” followed by “Cell cycle and DNA processing” and “Cellular communication/signal transduction mechanism” with 3.5, 3.3, 2.4 and 2.4 fold enrichment on the Sortin2-resistance interactome, respectively (Table 2). However, when more specific granular terms were analyzed, the results showed that the processes of “Regulation of DNA processing” and “Endocytosis” had the highest enrichment, with values of 11.1- and 8.3-fold, respectively (Additional file 7: Table S2). Moreover, the “Transcription elongation”, “Actin cytoskeleton” and “G2/M transition of mitotic cell cycle” processes were also highly enriched (more than 5-fold, Additional file 7: Table S2). Regarding the cell location of the Sortin2 resistance interactome members, CellLoc categorization showed that the highest over-represented categories were “Cytoskeleton”, “Transport vesicles” and “Punctate composite” (3.9-, 2.5- and 2.7-fold, respectively; Table 2). The enrichment of the “Cytoskeleton” category corresponded mainly to the “Actin cytoskeleton” category, which was enriched ninefold in the Sortin2-resistance interactome (Table 2).

**Table 2:** Functional and locational gene product categorization of the interactome network of genes whose deletion provokes resistance to Sortin2 in *S. cerevisiae*

	Representation on dataset			
	Sortin2 interactome (%)	Genome (%)	p-value	Enrichment in dataset (fold)
*Functional category*				
Cell fate	15.5	4.5	1.77E−13	3.5
Cell type differentiation	24.2	7.4	1.90E−19	3.3
Cell cycle and DNA processing	39.1	16.5	2.32E−20	2.4
Cellular communication/signal transduction mechanism	9.1	3.8	4.38E−05	2.4
Biogenesis of cellular components	31.1	14.0	6.35E−14	2.2
Cell rescue, defense and virulence	19.2	9.0	6.05E−08	2.1
Protein fate (folding, modification, destination)	38.0	18.8	1.55E−14	2.0
Interaction with the environment	15.2	7.6	7.12E−06	2.0
Protein with binding function or cofactor requirement (structural or catalytic)	27.5	17.1	6.42E−06	1.6
Cellular transport, transport facilities and transport routes	25.3	16.9	1.82E−04	1.5
Transcription	23.5	17.5	5.99E−03	1.3
*Locational category*				
Cytoskeleton	13.0	3.3	6.31E−13	3.9
Actin cytoskeleton	8.0	0.9	3.55E−16	9.0
Punctate composite	6.2	2.3	1.63E−04	2.7
Transport vesicles	5.8	2.3	5.50E−04	2.5
Cytoplasm	61.2	46.3	2.91E−07	1.3
Nucleus	46.3	34.8	3.46E−05	1.3

Category representation of the abundance within the Sortin2-resistance interactome network (dataset 273 genes) and the *S. cerevisiae* genome (6131 genes). The *p*-value was calculated using the hypergeometric distribution as the statistical test. *p* < 0.005 was considered as significant.

## Discussion

Using a reverse chemical genetics approach six *S. cerevisiae* mutants were identified that did not show a CPY sorting defect when treated with Sortin2. This reverse chemical screen confirmed the specificity of the effect of Sortin2 within the endomembrane system as suggested previously for genes identified in Sortin2 hypersensitive screen [14]. The most enriched biological process among Sortin2-resistant mutants was endocytosis, and importantly, this compound affects the speed of internalization of an endocytic tracer towards the vacuole. These results show the power and consistency of genetic screens to unravel the effects of a bioactive compound and its specificity.

Sortin2-resistant mutants treated with Sortin2 did not show either CPY mistargeting or enhancing of the endocytic pathway that was observed in the wild type. This genetic evidence suggests that the Sortin2 effects of trafficking to the vacuole through the secretory system and endocytosis are linked. The Sortin2 structure–activity relationship analysis consistently supports this connection, since the Sortin2 effect on endocytosis depends on the same chemical features (see below) that have been shown for CPY trafficking [14]. As the endocytic pathway converges on the same organelle, it is foreseeable that both observed phenotypes might be due to higher trafficking rate towards to vacuole at late endosome level. Sortin2-CPY secretion could be explained by lower abundance of CPY receptor at the TGN due to its higher anterograde trafficking rate. This is consistent with the faster traffic of FM4-64 endocytosis towards to vacuole due to Sortin2. We were unable to define precisely whether the alteration of both pathways shares the same molecular mode of action. Data from Sortin2-hypersensitive screen [14] together with the link between endocytosis and secretory pathway support that Sortin2 cellular target is located at late endosomal trafficking events.

Since *clc1*, *sla1* and *met18* resistance is observed at any tested chemical concentration the mode of action of Sortin2 could involve its binding to the missing gene product in the corresponding mutant. However the possibility that these proteins are involved in Sortin2 intracellular availability could not be rule out. In the case that the gene products interact with the bioactive molecule they may do so with the two part of Sortin2 since their loss of function mutants were resistant to 5537685 as well. In this scenario how Sortin2 affects both endocytic and secretory pathways is undetermined. Clc1 is a structural protein that allows generation of clathrin-coated vesicles (CCVs) from plasma membrane (PM) and TGN for endocytic and secretory pathways respectively [42]. Also Sla1 has a function in endocytic CCVs formation as is present at clathrin-coated pits at plasma membrane [43]. Interestingly it has been proposed a role of SLA1 in late endosome-to-vacuole trafficking likely related to clathrin function as well [44, 45]. Based on the close functional relationship between Clc1 and Sla1 it is plausible that Sortin2 is interacting with the entire structure at the clathrin pit enhancing vesicle trafficking. In addition Met18 genetically interacts with Sla1 suggesting a related function however still unknown [46].

On the other hand, it is unlikely that YJL175W, DFG10 and DPL1 gene products would correspond to the molecular targets of Sortin2 since its deletions mutants are sensitive to Sortin2 (20 μM and higher) on its effect on CPY trafficking. Likely in this case the lack of gene products induces a perturbation of endomembrane system which compensates the effect of Sortin2. Dfg10 participates in dolichol biosynthesis and its loss of function mutant has defects on CPY N-glycosylation [32] that could affect its secretion. Thus, Dfg10 deficiency could cause slower rate of CPY secretion therefore higher dose of Sortin2 is required to trigger the same effect. Furthermore the partial resistance of *dpl1* could be explained by compensation effect on protein trafficking. *dpl1* accumulates phytosphingosine-1-phosphate (PHS1P) which is the substrate Dpl1 metabolizes [35]. PHS1P has a signaling role in the cell affecting gene transcriptional level, which includes genes involved in protein sorting and targeting cellular process [47]. Therefore, the lack of Dpl1 could alter transcription levels of protein trafficking genes compensating Sortin2 effect over trafficking pathways mediates by PHS1P accumulation.

The physical and genetic interactome analysis revealed that one of the most enriched GO processes was “vesicular cellular import” (8.3-fold enrichment) in which all genes belong to the term “endocytosis”. This result supports our observations of FM4-64 internalization and confirms that most likely the still undetermined molecular targets of Sortin2 are related to the cellular trafficking pathways. Moreover, interactome GO component classification revealed that of all possible cellular components, only five locations had significant representation on the Sortin2-resistance dataset. Three of these are involved in or related to endomembrane system components; cytoskeleton, punctate composite and transport vesicles. The high representation of “DNA processing and transcription elongation GO function” is consistent with the presence of MET18 within genes whose deletion confers resistance to Sortin2. In addition, high representation of these cellular functions correlates with the GO component, showing that nuclear location is represented in the dataset. However how these types of GO molecular processes and MET18 function are related to protein trafficking still remains unclear. Interestingly, several proteins involved in the endocytic process have been also related to chromatin remodeling, transcription and cell division functions supporting that those processes are somehow related [48, 49]. These findings may provide an explanation for highly represented processes in our interactome analysis and support that Sortin2 is affecting mainly endocytosis and/or endocytic-related processes.

Analyzing the structure–bioactivity relationship of Sortin2 provided insight into the molecular mechanism of its mode of action. The Sortin2 analog that lacks the sulfonate group on Sortin2 causes no effect in the endocytosis time frame. Replacing the sulfonate group with a group with a dense electron cloud such the benzoic ring or carboxyl group restituted Sortin2 effect on endocytosis. However, compound 6269701 did not affect the endocytosis, showing the importance of the chloride in the chlorobenzene ring. It is possible that rather than chloride itself being important, the lack of bioactivity is due to steric hindrance of the nitro group. Therefore, the effect of Sortin2 on endocytosis depends on these two parts of the molecule, the sulfonate group and the chlorobenzene ring.

Interestingly, for both biological effects of Sortin2 on endomembrane trafficking, the same structural features of Sortin2 are required. For triggering the secretion of CPY, it was shown that both structural features of Sortin2, the sulfonate and the chlorobenzene ring are equally necessary [14]. Consistently, chemical 6269701 was also inactive for CPY trafficking [14]. Overall, the analysis of Sortin2 functional structure features strongly support a link between its effect on trafficking to the vacuole and the endocytic pathway.

Regarding Sortin2 as a tool for research, we would like to stress that this novel compound has a distinct biological value since it activates specifically the endocytic trafficking towards the vacuole while many discovered drugs block or inhibit cellular processes within endomembrane system. This compound is also very specific, affecting a restrictive set of cellular components. The power of using such a biological modulator has been proved by the extensive use of bioactive compounds in cell biology.

## Conclusions

Critical *S. cerevisiae* proteins for the mode of action of Sortin2 have been identified by means of reverse chemical-genetics approach. Endocytosis is the principal biological process targeted by Sortin2 base on mutant phenotypes, GO annotation and the interactome of the six ORFs whose deletion caused Sortin2 resistance. In fact Sortin2 treatment enhanced trafficking of the endocytic tracer FM4-64 toward the vacuole supporting the consistence of the genetic data. Overall the link of the Sortin2 effect of on the secretory pathway and its effect on endocytosis is supported by the genetic, cellular and chemical data in *S. cerevisiae.*

## Methods

### Chemical treatments

Sortin2 and its related structures were obtained from ChemBridge (San Diego, CA, USA); the latter are referred to here by the identification numbers assigned by the manufacturer. *S. cerevisiae* were grown in regular Yeast Peptone Dextrose (YPD) liquid or solid (1.5% agar). Single yeast colonies were picked from YPD-agar plates, inoculated in liquid YPD and grown for 48 h at 28°C with constant shaking to generate an initial culture for the assays. Chemical treatments were performed in medium supplemented with the designated compounds or 1% DMSO as a negative control. Cultures were grown with the chemical compound for 72 h at 28°C. To evaluate the effect of Sortin2 on yeast growth performance, Sortin2-resistant strain cultures at equal initial optical density (OD_600_ = 0.2) were grown in YPD supplemented with 20 µM Sortin2 or 1% DMSO at 28°C. Subsequently OD_600_ was measured at different times. For evaluating cell viability, a fraction of cells grown in these conditions for 72 h were diluted 1 to 100,000 and plated on YPD on triplicates. Colony-forming unit (CFU) were scored after 2 days. The assay was repeated twice.

### Sortin2 resistance screening

The primary screen was performed using the *S. cerevisiae* haploid deletion library that contains 4,800 yeast strains generated from the BY4742 parental strain (MATalpha *his3Δ1leu2Δ0lys2Δ0ura3Δ0*; Open Biosystems, Huntsville, AL, USA). The strains (OD_600_ = 0.2) were grown in darkness in YPD liquid medium supplemented with 47 µM Sortin2 and 1% DMSO, in a microplate format. After 72 h, growth medium was collected and analyzed for secreted CPY as described previously [10] using alkaline phosphatase labeled secondary antibody. All the deletion strains that did not secrete CPY due to Sortin2 in the primary screen were analyzed for CPY secretion at increasing concentrations of Sortin2 (5, 10, 20 and 40 μM). This assay was performed using peroxidase labeled secondary antibody to enhance its sensitivity and it was repeated three times. Mutants were considered as resistant to Sortin2 if CPY secretion was not detected at concentrations less than or equal to 10 μM because this was the concentration that trigger secretion of CPY in the wild type strain using peroxidase detection.

### FM4-64 endocytosis assay

Yeast cultures (OD_600_ = 0.2) were grown with 20 µM of chemical compound and 1% DMSO for 72 h at 28°C in darkness with constant shaking. Cell cultures were centrifuged at 5,000 rpm for 4 min and re-suspended in fresh growth medium. Cells were incubated with 24 μM FM4-64 (Invitrogen) at 4°C for 30 min. Subsequently, the FM4-64-containing medium was replaced with fresh YPD medium and cultures was incubated at 28°C. To observe FM4-64 distribution, 5 µl of the suspension were placed on a slide pretreated with 1 mg/ml Convanavalin A. Endocytosis of FM4-64 was examined at 0, 15, 25, 40 and 60 min at 28°C using Zeiss LSM 510 confocal microscope with a 543 nm emission filter. Approximately 25 cells were observed for each condition. The experiment was repeated three times; representative images are shown.

### Bioinformatic analysis

To retrieve information about gene annotation, gene products, gene ontology (GO) and mutant phenotypes of each ORF, the Saccharomyces Genome Database (SGD, http://www.yeastgenome.org/) was queried. The interactome network was obtained by submitting the query gene products to the OSPREY 1.2.0 software platform [41]. Function and localization of gene products of the interactome network were categorized using the FunCat and CellLoc classification systems from The Munich Information Center for Protein Sequences (MIPS, http://www.mips.gsf.de).

## Additional files

Additional file 1: Table S1. Saccharomyces deletion strains selected on the Sortin 2 primary screen. All the 36 deletion mutants selected as Sortin2 resistant in the primary screen are listed. They were tested on a secondary screen for Sortin2 resistance classifying them as according wild type behavior (Wt) and resistant to Sortin2 (R). Results on the secondary screen were judged based on the behavior of experimental repetitions (Number of repetitions). Additional file 2: Figure S1. Sortin2 short time treatment does not inhibit growth of Sortin2 resistant mutants. *S. cerevisiae* parental line (WT) and Sortin2 resistant mutants were grown on YPD supplemented with 1% DMSO (control) and YPD supplemented with 47 μM Sortin2 (Sortin2). Growth performance was evaluated by OD_600_ at different incubation times. The assay was repeated twice with experimental triplicates. Additional file 3: Figure S2. Sortin2 does not affect viability of Sortin2 resistant mutants. *S. cerevisiae* parental line (WT) and Sortin2 resistant mutants were grown on YPD supplemented with 1% DMSO (control) and YPD supplemented with 20 μM Sortin2 (Sortin2) for 72 h. Number of cells were evaluated by measuring OD_600_ (A). A fraction of cells in every condition were plated in YPD medium to analyze viability. The results were expressed as colony-forming unit (CFU) for every 10^8^ cells (B) as well as a percentage of viability (C). Statistical significance was evaluated with Student´s *t* test (**p* < 0.05, ***p* < 0.01). Additional file 4: Figure S3.
*sla1* is sensitive to three different CPY-secretion triggering compounds. Parental (WT) and *sla1* strains were grown in YPD medium supplemented with the indicated concentrations of Sortin2, furan, Brefeldin A and Endosidin1. The control condition (0 μM Sortin2) contained 1% DMSO. The presence of CPY was analyzed on the growth medium by dot-blot using a CPY monoclonal antibody. Additional file 5: Figure S4. Figure 3. Enhancing of endocytic trafficking depends on Sortin2 structural features. *S. cerevisiae* parental line was grown on YPD 1% DMSO (control) and YPD supplemented with 20 μM of different Sortin2-structural analogs. Afterwards cells were incubated with 24 μM FM4-64 for 30 min at 4°C. Then turned to 28°C to be imaged subsequently by confocal microscopy at different incubation times. Images of twenty-five min incubation are shown. Two images are representative of 20 cells. The experiment was performed more than 3 times. Scale bar represents 5 μm. Additional file 6: Figure S5. FM4-64 reaches the vacuole in Sortin2 resistant mutants. *S. cerevisiae* parental line (WT) and Sortin2 resistant mutants were grown on YPD (control) and YPD supplemented with 20 μM Sortin2. Cells were incubated with 24 μM FM4-64 for 30 min at 4°C. After 40 (A) and 60 (B) min at 28°C, cells were imaged by confocal microscopy. Images are representative of 20 cells. Scale bar represents 5 μm. Additional file 7: Table S2. Functional and locational gene product categorization of interactome network of genes whose deletion provokes resistance to Sortin2 in *S. cerevisiae*. Category representation of the abundance within the Sortin2-resistance interactome network dataset (273 genes) and the *S. cerevisiae* genome (6,131 genes). p < 0.005 was considered as significant.
