# Supplementary material for: Sortin2 enhances endocytic trafficking towards the vacuole in Saccharomyces cerevisiae
Source: Biol Res. 2015 Jul 25;48(1):39. doi: 10.1186/s40659-015-0032-9 (PMC4515019; doi:10.1186/s40659-015-0032-9)
Supplement: Additional file 1: Table S1. — Saccharomyces deletion strains selected on the Sortin 2 primary screen. All the 36 deletion mutants selected as Sortin2 resistant in the primary screen are listed. They were tested on a secondary screen for Sortin2 resistance classifying them as according wild type behavior (Wt) and resistant to Sortin2 (R). Results on the secondary screen were judged based on the behavior of experimental repetitions (Number of repetitions). [file 40659_2015_32_MOESM1_ESM.docx]

**Supplemental Table 1.**

| ORF | Mutant | Number of repetitions | Sortin2 sensitivity |
| --- | --- | --- | --- |
| YBL007C | SLA1 | 5 | R |
| YBL083C | YBL083C | 2 | Wt |
| YBR168W | PEX32 | 2 | Wt |
| YCR020-W | HTL1 | 2 | Wt |
| YDL101C | DUN1 | 2 | Wt |
| YDL222C | FMP45 | 2 | Wt |
| YDR055W | *PST1* | 2 | Wt |
| YDR294C | DPL1 | 3 | R |
| YGL004C | RPN14 | 2 | Wt |
| YGL058W | *RAD6* | 2 | Wt |
| YGR093W | YGR093W | 2 | Wt |
| YGR167W | CLC1 | 5 | R |
| YGR188C | BUB1 | 2 | Wt |
| YGR229C | SMI1 | 2 | Wt |
| YHR066W | SSF1 | 2 | Wt |
| YHR194W | MDM31 | 2 | Wt |
| YIL049W | DFG10 | 5 | R |
| YIL128W | MET18 | 5 | R |
| YJL0045W | YJL0045W | 2 | Wt |
| YJL139C | YUR1 | 2 | Wt |
| YJL175W | YJL175W | 3 | R |
| YJR121W | YJR121W | 2 | Wt |
| YKL107W | YKL107W | 2 | Wt |
| YKL126W | *YPK1* | 2 | Wt |
| YLR018C | *POM34* | 2 | Wt |
| YLR226W | *BUR2* | 2 | Wt |
| YML016C | *PPZ1* | 2 | Wt |
| YML034W | SRC1 | 2 | Wt |
| YML035C-A | YML035C-A | 2 | Wt |
| YMR014W | *BUD22* | 2 | Wt |
| YNR049C | MSO1 | 2 | Wt |
| YOL132W | GAS4 | 2 | Wt |
| YOR059C | YOR059C | 2 | Wt |
| YOR073W | SGO1 | 2 | Wt |
| YOR268C | YOR268C | 2 | Wt |
| YOR269W | PAC1 | 2 | Wt |
