# Supplementary figures and images for: Sortin2 enhances endocytic trafficking towards the vacuole in Saccharomyces cerevisiae
Source: Biol Res. 2015 Jul 25;48(1):39. doi: 10.1186/s40659-015-0032-9 (PMC4515019; doi:10.1186/s40659-015-0032-9)

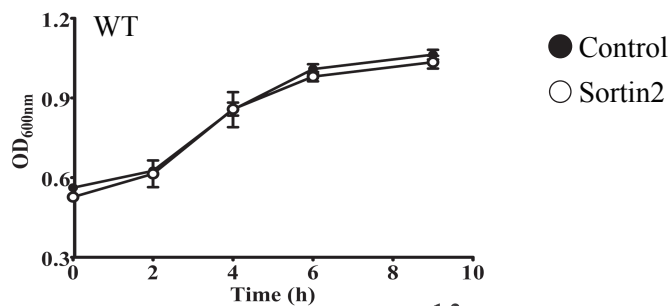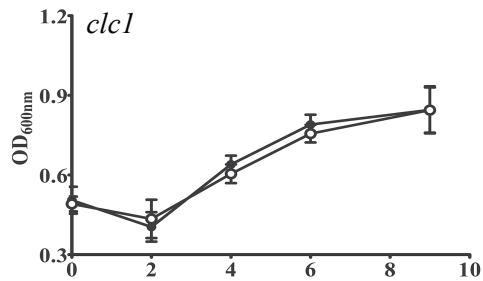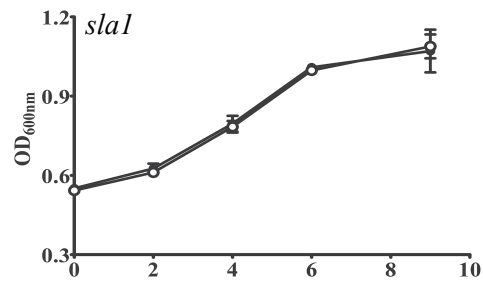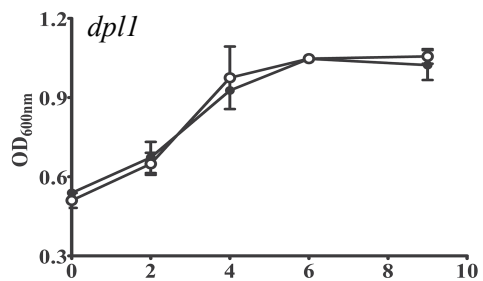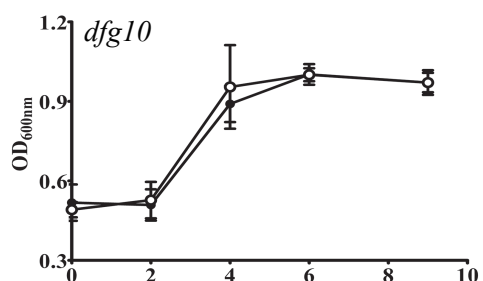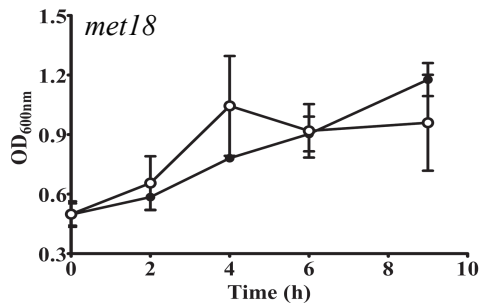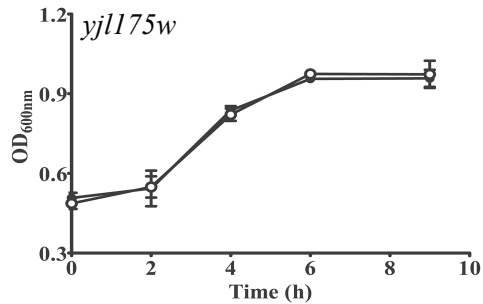

Supplement: Additional file 2: Figure S1. — Sortin2 short time treatment does not inhibit growth of Sortin2 resistant mutants. S. cerevisiae parental line (WT) and Sortin2 resistant mutants were grown on YPD supplemented with 1% DMSO (control) and YPD supplemented with 47 μM Sortin2 (Sortin2). Growth performance was evaluated by OD600 at different incubation times. The assay was repeated twice with experimental triplicates. [file 40659_2015_32_MOESM2_ESM.pdf]

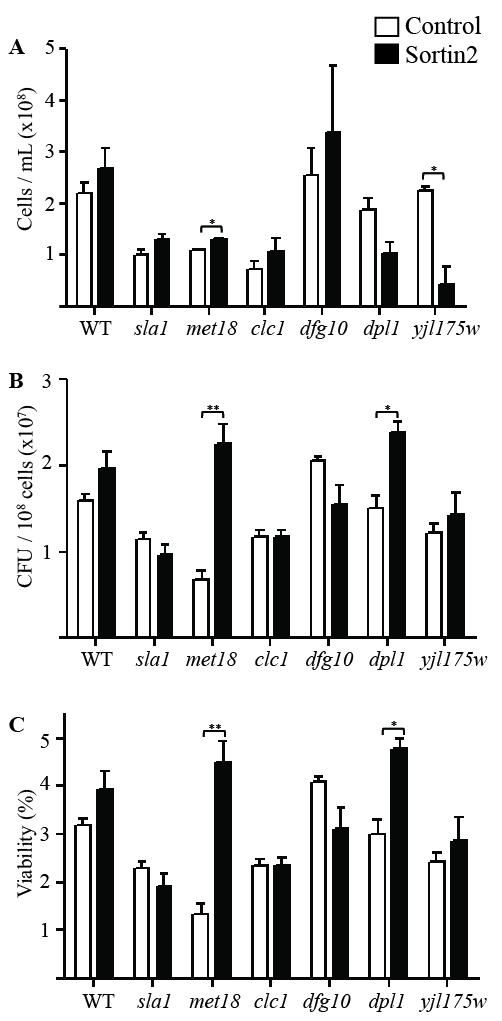

Supplement: Additional file 3: Figure S2. — Sortin2 does not affect viability of Sortin2 resistant mutants. S. cerevisiae parental line (WT) and Sortin2 resistant mutants were grown on YPD supplemented with 1% DMSO (control) and YPD supplemented with 20 μM Sortin2 (Sortin2) for 72 h. Number of cells were evaluated by measuring OD600 (A). A fraction of cells in every condition were plated in YPD medium to analyze viability. The results were expressed as colony-forming unit (CFU) for every 108 cells (B) as well as a percentage of viability (C). Statistical significance was evaluated with Student´s t test (*p < 0.05, **p < 0.01). [file 40659_2015_32_MOESM3_ESM.tiff]

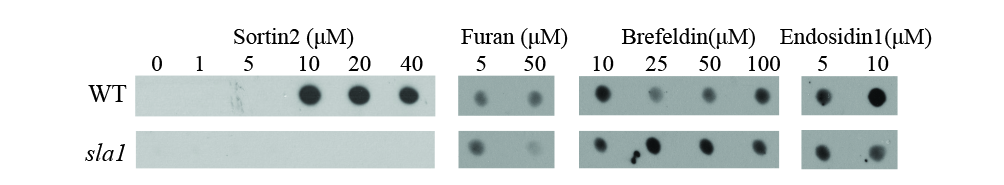

Supplement: Additional file 4: Figure S3. — sla1 is sensitive to three different CPY-secretion triggering compounds. Parental (WT) and sla1 strains were grown in YPD medium supplemented with the indicated concentrations of Sortin2, furan, Brefeldin A and Endosidin1. The control condition (0 μM Sortin2) contained 1% DMSO. The presence of CPY was analyzed on the growth medium by dot-blot using a CPY monoclonal antibody. [file 40659_2015_32_MOESM4_ESM.tiff]

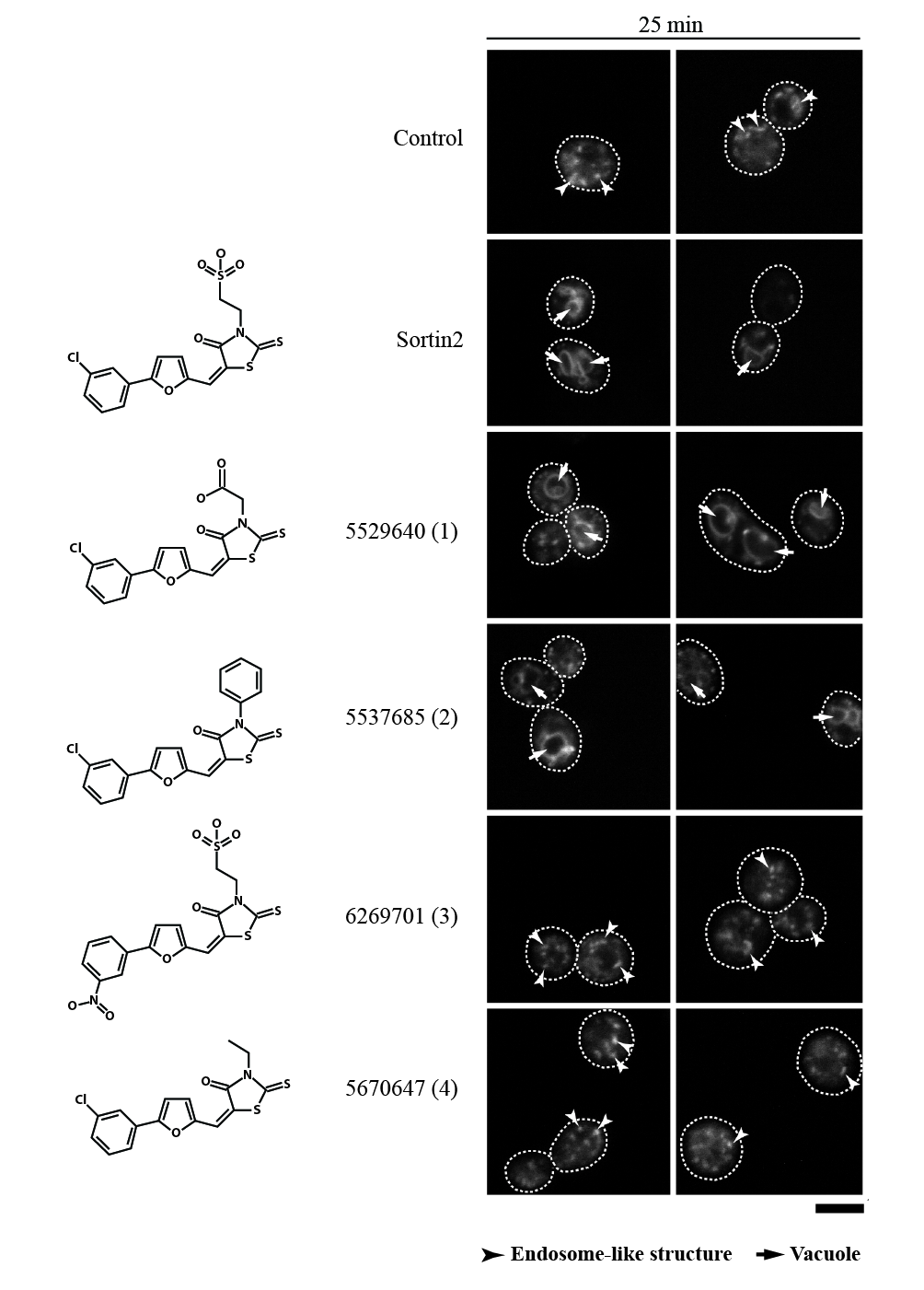

Supplement: Additional file 5: Figure S4. — Figure 3. Enhancing of endocytic trafficking depends on Sortin2 structural features. S. cerevisiae parental line was grown on YPD 1% DMSO (control) and YPD supplemented with 20 μM of different Sortin2-structural analogs. Afterwards cells were incubated with 24 μM FM4-64 for 30 min at 4°C. Then turned to 28°C to be imaged subsequently by confocal microscopy at different incubation times. Images of twenty-five min incubation are shown. Two images are representative of 20 cells. The experiment was performed more than 3 times. Scale bar represents 5 μm. [file 40659_2015_32_MOESM5_ESM.tiff]

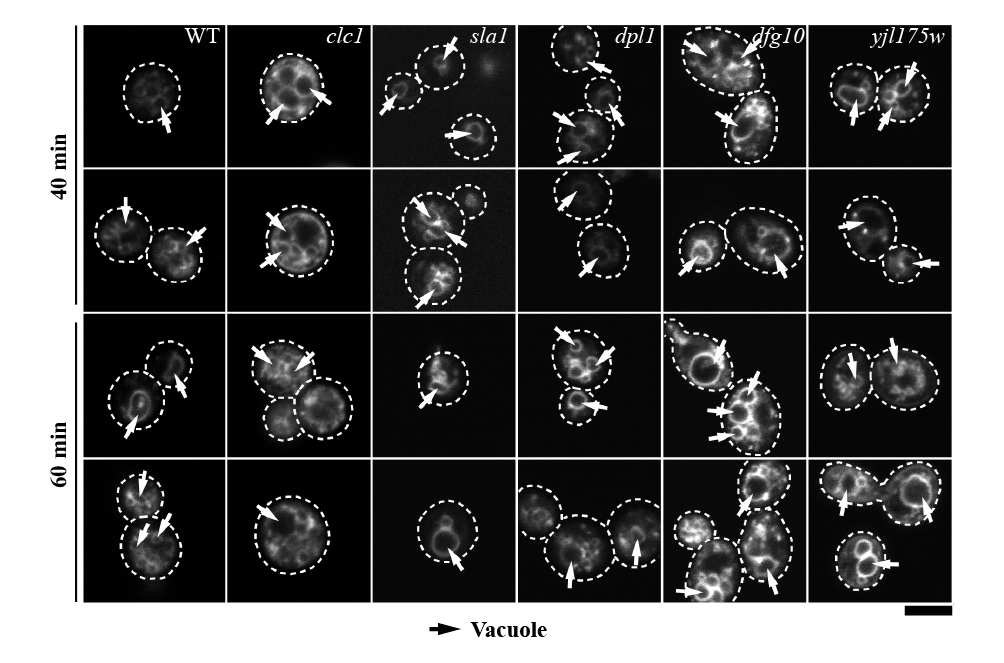

Supplement: Additional file 6: Figure S5. — FM4-64 reaches the vacuole in Sortin2 resistant mutants. S. cerevisiae parental line (WT) and Sortin2 resistant mutants were grown on YPD (control) and YPD supplemented with 20 μM Sortin2. Cells were incubated with 24 μM FM4-64 for 30 min at 4°C. After 40 (A) and 60 (B) min at 28°C, cells were imaged by confocal microscopy. Images are representative of 20 cells. Scale bar represents 5 μm. [file 40659_2015_32_MOESM6_ESM.tiff]
