# Supplementary material for: Sortin2 enhances endocytic trafficking towards the vacuole in Saccharomyces cerevisiae
Source: Biol Res. 2015 Jul 25;48(1):39. doi: 10.1186/s40659-015-0032-9 (PMC4515019; doi:10.1186/s40659-015-0032-9)
Supplement: Additional file 7: Table S2. — Functional and locational gene product categorization of interactome network of genes whose deletion provokes resistance to Sortin2 in S. cerevisiae. Category representation of the abundance within the Sortin2-resistance interactome network dataset (273 genes) and the S. cerevisiae genome (6,131 genes). p < 0.005 was considered as significant. [file 40659_2015_32_MOESM7_ESM.docx]

**Supplemental Table 2.**

|  | | **REPRESENTATION ON DATASET** | |  |  |
| --- | --- | --- | --- | --- | --- |
| **FUNCTIONAL CATEGORY** | | **Sortin2 Interactome (%)** | **Genome (%)** | **p-value** | **Enrichment on dataset**  **(-fold)** |
|  |  | |  |  |  |
| Cell fate | 15.5 | | 4.5 | 1.77E-13 | 3.5 |
| Cell growth/morphogenesis | 14.1 | | 3.9 | 7.42E-13 | 3.6 |
| Cell type differentiation | 24.2 | | 7.4 | 1.90E-19 | 3.3 |
| Fungal / microorganism cell type differentiation | 24.2 | | 7.4 | 1.09E-19 | 3.3 |
| Fungal and other eukaryotic cell type  differentiation | 24.2 | | 7.4 | 1.09E-19 | 3.3 |
| Budding, cell polarity and filament formation | 19.5 | | 5.1 | 1.18E-18 | 3.8 |
| Development of ascobasidio- or xygospora | 7.6 | | 2.7 | 1.62E-05 | 2.8 |
| Cell cycle and DNA processing | 39.1 | | 16.5 | 2.32E-20 | 2.4 |
| DNA processing | 23.5 | | 8.5 | 5.33E-15 | 2.8 |
| DNA syntesis and replication | 6.2 | | 2.3 | 1.36E-04 | 2.7 |
| Extension/polymerization activity | 2.9 | | 0.6 | 1.88E-04 | 4.8 |
| DNA recombination and DNA repair | 10.5 | | 4.1 | 2.35E-06 | 2.6 |
| DNA repair | 8.0 | | 2.6 | 2.05E-06 | 3.1 |
| DNA restriction or modification | 11.9 | | 3.2 | 3.68E-11 | 3.7 |
| DNA conformation modification (e.g. chromatin) | 11.5 | | 3.1 | 3.17E-11 | 3.8 |
| Regulation of DNA processing | 1.4 | | 0.1 | 2.44E-04 | 11.1 |
| Cell cycle | 25.3 | | 10.6 | 9.94E-13 | 2.4 |
| Mitotic cell cycle and cell cycle control | 17.3 | | 7.3 | 7.39E-09 | 2.4 |
| Mitotic cell cycle | 8.7 | | 2.7 | 2.68E-07 | 3.2 |
| G2/M transition of mitotic cell cycle | 4.3 | | 0.8 | 1.43E-06 | 5.4 |
| Meiosis | 6.2 | | 2.6 | 8.56E-04 | 2.3 |
| Cytokinesis (cell divisio)/septum formation and  hydrolysis | 4.7 | | 1.2 | 1.31E-05 | 4.1 |
| Cellular communication/ signal transducción mechanism | 9.1 | | 3.8 | 4.38E-05 | 2.4 |
| Cellular signalling | 8.7 | | 3.2 | 8.21E-06 | 2.7 |
| Enzyme mediated signal transduction | 5.8 | | 2.2 | 2.83E-04 | 2.7 |
| Protein kinase | 4.0 | | 0.9 | 3.66E-05 | 4.3 |
| Biogenesis of cellular components | 31.1 | | 14.0 | 6.35E-14 | 2.2 |
| Cytoskeleton/structural proteins | 17.3 | | 4.1 | 1.94E-18 | 4.2 |
| Actin cytoskeleton | 9.4 | | 1.6 | 3.71E-14 | 6.0 |
| Nucleus | 6.15 | | 2.43 | 3.47E-04 | 2.5 |
| Organization of chromosome structure | 5.4 | | 1.5 | 9.04E-06 | 3.7 |
| Cell rescue, defense and virulence | 19.2 | | 9.0 | 6.05E-08 | 2.1 |
| Stress response | 17.3 | | 7.3 | 9.21E-09 | 2.4 |
| Osmotic and salt stress response | 4.7 | | 1.0 | 1.48E-06 | 4.9 |
| Protein Fate (folding, modification, destination) | 38.0 | | 18.8 | 1.55E-14 | 2.0 |
| Protein folding and stabilization | 3.6 | | 1.5 | 8.63E-03 | 2.4 |
| Protein Modification | 21.3 | | 10.0 | 8.46E-09 | 2.1 |
| Modification by phosphorilation,  dephosphorilation, autophosphorilation | 9.4 | | 3.0 | 1.88E-07 | 3.1 |
| Modification by acetylation, deacetylation | 5.4 | | 1.1 | 2.74E-07 | 4.8 |
| Assembly of protein complexes | 10.1 | | 3.2 | 5.33E-08 | 3.1 |
| Interaction with the enviroment | 15.2 | | 7.6 | 7.12E-06 | 2.0 |
| Cellular sensing and response to external stimulus | 11.5 | | 4.6 | 1.09E-06 | 2.5 |
| Chemoperception and response | 8.7 | | 3.9 | 1.78E-04 | 2.2 |
| Pheromone response, mating-type  determination, sex-specific proteins | 8.0 | | 3.1 | 3.46E-05 | 2.6 |
| Protein with binding function or cofactor requirement (structural or catalytic) | 27.5 | | 17.1 | 6.42E-06 | 1.6 |
| Protein Binding | 15.5 | | 6.4 | 2.71E-08 | 2.4 |
| Cellular transport, transport facilities and transport routes | 25.3 | | 16.9 | 1.82E-04 | 1.5 |
| Transport routes | 21.0 | | 11.6 | 3.43E-06 | 1.8 |
| Cellular import | 10.5 | | 2.4 | 1.28E-11 | 4.3 |
| Vesicular cellular import | 8.0 | | 1.0 | 2.08E-15 | 8.3 |
| Endocytosis | 8.0 | | 1.0 | 2.08E-15 | 8.3 |
| Transcription | 23.5 | | 17.5 | 5.99E-03 | 1.3 |
| RNA synthesis | 21.0 | | 10.3 | 6.28E-08 | 2.0 |
| mRNA synthesis | 20.2 | | 9.4 | 1.30E-08 | 2.2 |
| General transcription activities | 10.8 | | 3.8 | 1.62E-07 | 2.8 |
| Transcription elongation | 3.3 | | 0.5 | 5.62E-06 | 6.5 |
| Transcriptional control | 18.4 | | 8.1 | 9.58E-09 | 2.3 |
